# Supplementary material for: Perceptual visual dependence for spatial orientation in patients with schizophrenia
Source: PLoS One. 2022 Dec 1;17(12):e0278718. doi: 10.1371/journal.pone.0278718 (PMC9714874; doi:10.1371/journal.pone.0278718)
Supplement: Attachment — Submitted filename: Response to Reviewers.docx [file pone.0278718.s002.docx]

**Response to Editor**

1. Please ensure that your manuscript meets PLOS ONE's style requirements, including those for file naming.

The authors confirm that the submitted manuscript meets PLOS ONE’s requirements including those for file naming.

2. Please provide additional details regarding participant consent. In the ethics statement in the Methods and online submission information, please ensure that you have specified what type you obtained (for instance, written or verbal, and if verbal, how it was documented and witnessed). If your study included minors, state whether you obtained consent from parents or guardians. If the need for consent was waived by the ethics committee, please include this information.

We have included the following paragraph in the manuscript detailing how informed consent was obtained: “After the aim and impact of the study were explained to the healthy controls and to the patients and their relatives, informed verbal consent was taken. An impartial witness, who was not a member of the study team and worked at the center where the patients were treated, endorsed that the consent from patients and their relatives was voluntary and freely given. Only those who volunteered were included in the study”.

3. In your Data Availability statement, you have not specified where the minimal data set underlying the results described in your manuscript can be found. PLOS defines a study's minimal data set as the underlying data used to reach the conclusions drawn in the manuscript and any additional data required to replicate the reported study findings in their entirety. All PLOS journals require that the minimal data set be made fully available.

Upon re-submitting your revised manuscript, please upload your study’s minimal underlying data set as either Supporting Information files or to a stable, public repository and include the relevant URLs, DOIs, or accession numbers within your revised cover letter. For a list of acceptable repositories, please see <http://journals.plos.org/plosone/s/data-availability#loc-recommended-repositories>. Any potentially identifying patient information must be fully anonymized.

Important: If there are ethical or legal restrictions to sharing your data publicly, please explain these restrictions in detail. Please see our guidelines for more information on what we consider unacceptable restrictions to publicly sharing data: <http://journals.plos.org/plosone/s/data-availability#loc-unacceptable-data-access-restrictions>. Note that it is not acceptable for the authors to be the sole named individuals responsible for ensuring data access.

We will update your Data Availability statement to reflect the information you provide in your cover letter.

The minimal data set can be found in an Excel file uploaded as Supporting Information File.

4. Please review your reference list to ensure that it is complete and correct. If you have cited papers that have been retracted, please include the rationale for doing so in the manuscript text, or remove these references and replace them with relevant current references. Any changes to the reference list should be mentioned in the rebuttal letter that accompanies your revised manuscript. If you need to cite a retracted article, indicate the article’s retracted status in the References list and also include a citation and full reference for the retraction notice.

There were no citations of papers that have been retracted. However additional citations were included to further support the Discussion section.

**Response to Reviewers**

The authors would like to thank both reviewers for their comments which enhanced the quality of the manuscript. The authors accommodated all comments as best as they can.

**Reviewer #1:**

The manuscript is very well written and states a very useful finding about perceptual differences in individuals with psychosis. Every question/concern that came to mind while I was reading the manuscript was answered/addressed in the Discussion, so I have very few suggestions for improvement for this very excellent manuscript.

My two small suggestions are:

-- Perhaps the statement "probably indicating a sub-clinical vestibular dysfunction" should be removed from the abstract, or changed to a statement like "consistent with previous reports of possible vestibular dysfunction in patients with schizophrenia" since there are no measurements of vestibular function in the present work.

We took the reviewer’s suggestion and changed the statement according to their recommendation.

-- when I saw the data in Figure 3, I assumed (at first -- until I read the discussion) that this correlation was driven by age. The lack of correlation in controls is a key finding for supporting the conclusion that the correlation of SVV with disease duration is not simply because visual/vestibular integration is impaired with age. Perhaps the authors could include a 2nd panel in Fig. 3 showing the lack of correlation in the control population? This might be a very convincing visualization.

We included an additional figure depicting the effect of ageing on visual dependence in both healthy controls and patients (2 graphs).

--------------------------------------------------------------------------------------------------------

**Reviewer #2:**

- RDT and VD explanations are well written. But I suggest the authors to group the first 3 paragraphs together for more coherence.

The authors thank the reviewer for this suggestion, but they felt that it was sufficient to group the first two paragraphs only, so as not to make it into a very long paragraph.

- Line 76: The authors mention few articles on vestibular dysfunction in schizophrenia. The only one cited is from the 1970s. It would be wise to put one or two more recent references to support this argument.

The authors added a relatively more recent reference: “Haghgooie S, Lithgow B J, Gurvich C, Kulkarni J. Quantitative detection and assessment of schizophrenia using electrovestibulography. 4th International IEEE/EMBS Conference on Neural Engineering, 2009; 486 – 489. doi: 10.1109/NER.2009.5109339.

- Line 83: “This deficit may manifest as impaired functioning in dynamic visual environments.” Can you give some examples that are more meaningful?

The authors provided some real-life examples such as crowded or busy environments, as walking in supermarket aisles, movements of crowds or traffic, moving images at the cinema, trees swaying, or while driving fast on a highway.

- Line 112: “All participants had normal or corrected-to-normal visual acuity.” Please specify the scale.

The authors ensured that the participants had 20/20 visual acuity, and participants were allowed to put on their corrective spectacles if necessary. This was added in the Methods section.

- Line 138: “Subjects observed the screen while sitting upright on an armless chair”. What was the distance measured between the screen and the eyes of the patient? And why? Was this distance the same for everyone?

According to many studies on RFT and RDT, the distance between the screen and the eyes of the participant is 80 cm. The center of the screen was at eye level of the observer. This was added in the Methods section. Yes, the distance was the same for all participants, as they were all tested on the same computer/screen set up and the same place in the Psychiatry hospital.

- Line 162: “Participants were informed of the importance of spatial accuracy, and that the trials were not time restricted”. It would have been interesting to see how quickly the participants would have considered the task successful. Why didn't you measure this parameter?

The authors thank the reviewer for pointing this out. Fortunately, the software does record and stores the time of rod alignment for each trial. The authors analysed the time-related data and included the findings in the Results section. The significance of these results was included in the Discussion.

- Line 279: Regarding conflicting reports on the effect of ageing, don't you think that more recent articles have more legitimacy in the results? Also, did these articles use exactly the same task as yours? If no, how can you discuss with these results?

The authors did add more recent references in the Discussion on the effect of ageing on visual dependence, especially those utilizing the common and similar tests of visual dependence, specifically the RFT and the RDT.

- The effect of gender was not discussed even though men represent nearly 75% of the sample. It is thus likely that the gender effect is significant. Was it included in your analyses?

The authors appreciate this recommendation from the reviewer. Upon their suggestion, gender analysis was included, and the findings were addressed, with added references to enrich the Discussion.

- Do you have prospects for future research to address or possibly compensate for your limitations?

Definitely. The authors would like in future studies to add assessment of peripheral vestibular system function in patients with long-duration schizophrenia and determine any possible interference of long-term use of neuroleptic medications on vestibular functions.
