# Supplementary material for: Perceptual visual dependence for spatial orientation in patients with schizophrenia
Source: PLoS One. 2022 Dec 1;17(12):e0278718. doi: 10.1371/journal.pone.0278718 (PMC9714874; doi:10.1371/journal.pone.0278718)
Supplement: Attachment — Submitted filename: Response to Reviewers.docx [file pone.0278718.s003.docx]

**Response to reviewers**

Once again, the authors would like to thank the editor and the two reviewers for improving the quality of this manuscript.

**Reviewer #1:**

My few concerns from the previous round of review have been addressed. On re-reading the abstract I noticed that on the first use of SVV when it is defined it is defined as perceived visual vertical instead of subjective visual vertical, which might be mildly puzzling to readers; just a tiny change you might think about making

We have substituted the word “perceived” by “subjective” in the abstract.

Reviewer #2: Dear authors,

I take into account the fact that you carried out a real work of bibliography following my remarks on the manuscript. I consider that your work to date is well done, in particular with the addition of recent bibliographic research as well as concrete examples illustrating your words. I transmitted to PLOS ONE the acceptance of the manuscript.

We, the authors, would like to thank the reviewer for their encouragement and confidence in the changes made in the first revision of the manuscript.

These last comments will help you to finalize your manuscript =

- Line 113 = I think I was misunderstood. I expect that the participants have normal or corrected-to-normal visual acuity. However, it would be wise to clarify the name of the scale used to measure the visual acuity. Is it a Snellen chart? Is it a Monoyer scale? Please clarify. I think this is an important criterion if other authors would like to follow your methodology.

We did add that the scale used to measure the visual acuity is the Snellen chart.

- Line 139 = Indeed, most visual studies mention eye-screen distances between 40cm and 100cm depending on the projection of the different stimuli. In relation to your biographical research, your answer is judicious.

We thank the reviewer for this encouraging comment.

- Line 328 = I see that my point about age and gender effect has been perfectly addressed in the manuscript, with solid argumentation based on recent literature. This is a very good point.

We thank the reviewer for this encouraging comment.

- Line 344 = Similarly, the remark about the time of rod alignment for each trial and also the time-related data were added in the results as well as the discussion and confronted with the literature. However, although the results were not significant between the two groups, I do not think it is necessary to comment on this.

We did remove from the Discussion section the paragraph where we attempted to provide a reason for the absence of group difference in time of rod alignment.

- Line 351 and line 369 = I find that the 2 paragraphs dealing with the effect of age on visual dependence are particularly well discussed. You compare the results currently reported in the article with those in the literature, and it's great.

We thank the reviewer for this encouraging comment.

- Line 372 = Finally, it would have been wise to specify the number of people recruited in the study of Ashish. I also appreciated that you compared it with your current article by indicating age ranges and specifying the age range of your population.

We included the number of people recruited (82 healthy adult volunteers) in the study of Ashish.
